# Fatty acids in filamentous pathogenic fungi: Multidimensional regulation and pathogenic mechanisms

**DOI:** 10.1080/21505594.2025.2561826

**Published:** 2025-09-15

**Authors:** Haiyan Lin, Yuejin Peng

**Affiliations:** a School of Biological and Chemical Engineering, Zhejiang University of Science and Technology, Hangzhou, China; b College of Plant Protection, Yunnan Agricultural University, Kunming, Yunnan, China

**Keywords:** Fatty acids, filamentous pathogenic fungi, virulence, organellar metabolism, host-pathogen interaction, regulatory networks

## Abstract

Fatty acids (FAs) metabolism constitutes a central regulatory network in the life activities of filamentous pathogenic fungi. This review systematically outlines the multifaceted roles of FA metabolism in filamentous pathogenic fungi, encompassing growth, development, pathogenicity, and environmental adaptation. FAs serve not only as fundamental structural components of cellular membranes and central units in energy metabolism, but also function as signaling molecules that regulate fungal morphogenesis, virulence factor expression, and host interactions. This work critically analyzes the dynamic equilibrium between FA synthesis and β-oxidation pathways, highlighting the hub functions of mitochondria and peroxisomes in energy supply, reactive oxygen species (ROS) metabolism, and virulence regulation. Furthermore, FAs coordinate lipid droplet dynamics, autophagic processes, and membrane transport through organellar systems (vacuoles, endoplasmic reticulum) while mediating host immune evasion. Finally, how the integrated FA metabolic networks and epigenetic regulation collectively influence fungal pathogenicity was explored. This review provides theoretical insights for overcoming significant challenges in controlling pathogenic fungi targeting FA metabolism.

## Introduction

Lipid metabolism is pivotal in the life cycle of filamentous pathogenic fungi, supporting essential cellular functions and serving as a crucial mediator of pathogenicity. Fatty acids (FAs), as key structural units and signaling precursors, dynamically reprogram fungal growth, development, virulence, and environmental adaptability. The significance of FA metabolism in host-pathogen interactions is increasingly recognized, as it forms part of a complex “lipid language” mediating communication between host and fungi. FAs sustain energy metabolism through synthesis and β-oxidation. Crucially, β-oxidation occurs primarily in mitochondria and peroxisomes – organelles essential for pathogenesis that also house energy supply and ROS metabolism [1,2]. This metabolic flexibility, including the utilization of host lipids as carbon sources, is vital for successful colonization and infection [3].

Structurally, lipids are fundamental for maintaining cell membrane homeostasis, a critical determinant of pathogenicity [4]. FA metabolism contributes to sterol and phospholipid synthesis. This establishes the foundation for infection by regulating two key factors: membrane fluidity and effector protein anchoring efficiency [5–8]. Furthermore, FA derivatives play vital signaling roles. For example, sphingolipids (e.g. ceramide, sphingosine-1-phosphate) are master regulators of membrane raft integrity, dimorphism, stress response, and overall pathogenicity in both dimorphic and filamentous fungi [9,10]. Oxylipins, which are derived from FAs, act as potent signaling molecules. They regulate pathogenic pathways and fungal development [11]. The sophisticated integration of FA metabolism, structure, and signaling in fungal virulence is underscored by the precise regulation of lipid composition. This regulation operates through distinct mechanisms. Primarily, transcription factors like HapX orchestrates conidial FAs homeostasis, ensuring cytomembrane functionality and stress resistance during infection initiation [12]. Additionally, novel lipases (e.g. those with the AHSMG motif) are potentially involved in lipid acquisition, membrane remodeling, and signaling during infection [13–15].

This review presents the first systematic analysis of FAs functional roles in filamentous pathogenic fungi, examining their multidimensional dynamics across biological timelines. This review integrates FA synthesis/degradation pathways, their dynamic cellular functions, and regulatory networks governing fungal growth, development, virulence, and stress resistance. This synthesis establishes FA metabolism as a central hub in host-pathogen interactions. The work comprehensively summarizes research spanning FAs biology to pathogenic mechanisms, offering new theoretical insights for pathogenic fungus control or utilization.

## Advances in FA synthesis and metabolism in filamentous fungi

### FA biosynthesis pathways and regulatory mechanisms

FA synthesis is a complex process involving multiple enzymes and regulatory factors. In filamentous fungi, this pathway primarily includes the generation of acetyl-CoA, the synthesis of malonyl-CoA, and the elongation and modification of FAs. Recent studies indicate that key enzymes and regulatory factors in this pathway are conserved across different filamentous fungi but exhibit distinct variations. For instance, in *Magnaporthe oryzae*, the acyl-CoA binding protein (MoAcb1) is implicated in regulating conidiation, appressorium development, and pathogenicity [16]. Additionally, the glyoxylate cycle is significant in FA metabolism. In *Sclerotinia sclerotiorum*, the absence of peroxisomal carnitine acetyltransferase (Pth2) impacts sclerotia development and oxalic acid accumulation [17]. Citrate-malate carriers (MaCT1/MaCT2/MaTCT) in the oleaginous fungus *Mortierella alpina* transport citrate and malate, providing substrates for FA synthesis. Overexpression of these carriers significantly increases FA accumulation [18].

### FA β-oxidation and other metabolic pathways

FA β-oxidation, primarily occurring in peroxisomes and mitochondria, is crucial for the catabolism of FAs. Recent studies highlight that key enzymes in this pathway are essential for the growth, development, and pathogenicity of filamentous fungi. For example, in *M. oryzae*, enoyl-CoA hydratase (Ech1) is vital for spore germination and mycelial viability [19]. The transcription factor *MoMsn2* orchestrates invasive hyphal growth through transcriptional regulation of *MoDCI1* (encoding dienoyl-CoA isomerase), which is involved in modulating β-oxidation flux and lipid droplet mobilization [20]. Additionally, other metabolic pathways of FAs, such as phospholipid synthesis and metabolism, are crucial in the physiological processes of filamentous fungi. In *Aspergillus fumigatus*, the absence of phospholipid methyltransferase (ChoC) compromises cell membrane integrity and reduces pathogenicity [21]. Notably, the *Aspergillus nidulans* acyl-CoA dehydrogenase ScdA, a pivotal catalytic component in the β-oxidation cascade, is strictly required for cellular utilization of short-chain FAs (e.g. butyric acid and caproic acid), with its deletion resulting in complete loss of butyryl-CoA dehydrogenase activity [22].

Furthermore, modifications of FAs, such as hydroxylation [23] and methylation [24], are critical components of FA metabolism. These modifications alter the physicochemical properties of FAs, affecting their cellular functions and consequently influencing fungal growth, development, and pathogenicity.

## Multiple roles of FAs in cellular organelles of filamentous fungi

### FA metabolism in mitochondria

Mitochondrial fatty acid (FA) metabolism constitutes a crucial control center governing the pathogenicity of filamentous fungi. By maintaining a dynamic equilibrium between the β-oxidation pathway and metabolic intermediate signaling, it orchestrates both invasive behavior and host adaptability. Critically, studies demonstrate that mitochondrial β-oxidation extends beyond its role as an energy-generating engine. For example, in *M. oryzae*, the deficiency of enoyl-CoA hydratase reduces conidial germination rate by 25%, and lowers the mechanical penetration force of the appressoria [19]. Furthermore, metabolic intermediates derived from FA metabolism directly enable diverse biological functions. Exogenous acetate disrupts the β-oxidation pathway in *Ustilago maydis*, inducing collapse of the mitochondrial membrane potential and triggering a ROS burst that activates programmed cell death [25]. Similarly, carnitine acetyltransferase (Pth2), which catalyzes acetyl-CoA transfer into mitochondria for further metabolism, is essential for host colonization by *S. sclerotiorum* [17]. The functional significance of mitochondrial integrity is further underscored by the mitochondrial BCS1 protein in *Beauveria bassiana*, which facilitates mitochondrial function and FA homeostasis, thereby directly linking mitochondrial roles to fungal virulence [26]. In *A. nidulans*, the *acuH* gene plays a critical role in acetyl-CoA transport during long-chain FA and acetic acid metabolism. Its transcription is upregulated when acetic acid or oleic acid serves as the carbon source, whereas strongly suppressed grown on glucose carbon source [27].

Collectively, this research not only delineates the regulatory principles of mitochondrial FA metabolism across energy production, signaling, and structural maintenance, but also elucidates the mechanisms underpinning mitochondrial microenvironment homeostasis during pathogenesis.

### Peroxisome’s role in FAs degradation and virulence

Peroxisomes are multifunctional eukaryotic organelles implicated in various functions like FA β-oxidation, detoxification of ROS, and glycolysis regulation. As an ancient and conserved regulatory hub of lipid metabolism in filamentous pathogenic fungi [28], peroxisomes contribute to fungal growth, environmental adaptation, and virulence. Genetic disruption of peroxisomal components profoundly impacts FA metabolism and fungal physiology. For instance, deletion of Pex13, Pex14, Pex14/17, and Pex7 impairs peroxisome biogenesis, leading to defective growth on FA substrates, reduced sporulation, and compromised appressorium formation and infectivity [29–31]. A Pex2/Pex12 defect in wheat scab interrupts the synthesis of deoxynivalenol (DON toxin) and results in a complete loss of pathogenicity [32]. Structurally, peroxisomal membrane lipid composition is essential for maintaining woronin body integrity. Perturbations in this system compromise hyphal septal sealing, exacerbate cytoplasmic leakage, and impair fungal development [33,34]. The Pex22 homolog in *Colletotrichum orbiculare* (melon anthracnose fungus) exemplifies this mechanism, where its deletion destabilizes woronin body anchoring, reduces appressorial turgor pressure, and eliminates host penetration capacity [35]. In *M. oryzae*, peroxisomal proteins MoPex1 and MoPex22 jointly regulate FA metabolism and cell wall integrity, with their deletion significantly attenuating infection capability [36,37].

Unlike their animal counterparts, the peroxisome in fungi serves as the primary site for long-chain FA degradation. Its metabolite, acetyl-CoA, not only provides energy for the mechanical penetration of infectious structures like the appressorium of *M. oryzae* [1] but also acts as a precursor for secondary metabolite synthesis. Coordinated metabolic regulation is exemplified in *A. nidulans* and *M. oryzae*, where FarA/FarB (Far1/Far2) transcription factors synchronize β-oxidation (*MFP1, FOXA*) and peroxisome biogenesis (*PEX6, PEX11*) genes to control conidial germination and host colonization [38,39]. In *A. nidulans*, the β-oxidation pathway involves AoxA, a peroxisomal long-chain fatty acyl-CoA oxidase essential for energy production from FAs. Notably, *AoxA* deletion mutants exhibit severely impaired growth on long-chain FA substrates [40]. Complementary to this system, a suite of fatty acyl-CoA synthetases (FatA-D, FaaA-B) has been identified as crucial mediators of FA degradation within peroxisomes [41].

In host-pathogen interactions, pathogenic fungi utilize peroxisomes to break down host cuticular lipids for carbon sources (e.g. corn smut fungus relies on β-oxidation to utilize host FAs), and lipid transport mediated by peroxisomes also influences the virulence of these fungi. During lipid transport to peroxisomes, the widely accepted pathway involves SCP2 being recruited by the peroxisomal matrix protein transporter Pex5, facilitating lipid transport to peroxisomes [42]. SCP2, a soluble nonspecific lipid transport protein, is capable of transporting FAs, phospholipids, and sterols. Besides lipid transport, SCP family proteins also trigger the biosynthesis of cholesterol [43]. Disruption of lipid transport through SCP2 reduces the germination capability of the spores, thereby reducing the efficiency of infecting insects [44]. In *U. maydis*, SCP2 has also been proven to be involved in the formation of virulence-dependent appressoria [45]. The evidence forms a cascade regulatory pathway based on peroxisomes that encompass FA breakdown, lipid transport, availability of energy, and virulence.

### The role of FAs in vacuolar degradation of cellular materials and endoplasmic reticulum

Vacuoles, as metabolic hubs in filamentous pathogenic fungi, play a crucial role in regulating the dynamic balance of FA and lipid metabolism, profoundly influencing fungal growth, development, environmental adaptation, and pathogenicity. In terms of nutrient storage and utilization, vacuoles store and degrade lipid droplets, providing essential energy for fungal invasion of hosts. For example, in the insect pathogenic fungus *B. bassiana*, the calcium-binding protein Caleosin maintains lipid homeostasis in spores by regulating lipid droplet dynamics. Its absence leads to delayed spore germination and significantly reduced pathogenicity [46]. Similarly, *Metarhizium robertsii* initiates the microautophagy of lipid-droplet (LD) degradation (microlipophagy) early in infection for rapid release of FAs, supplying energy for host cuticle penetration [47]. Further studies have demonstrated that vacuole-mediated autophagy is not only involved in lipid recycling but also directly linked to virulence through the regulation of secondary metabolite synthesis. In the maize pathogen *Fusarium verticillioides*, the absence of autophagy-related proteins FvAtg4/FvAtg8 leads to vacuolar dysfunction, lipid peroxidation, and inhibited fumonisin synthesis, ultimately reducing pathogenicity [48]. Additionally, vacuole-dependent membrane transport systems (e.g. COPII component MoSec24B) coordinate the fusion of autophagosomes with vacuoles, facilitating appressoria formation and host penetration in rice blast fungus *M. oryzae* [49].

In pathogenicity-related signal regulation, lipid signaling molecules on vacuolar membranes (such as phosphoinositide isomers) may recruit effector proteins or regulate membrane dynamics to influence host interactions. For instance, in wheat Fusarium head blight fungus *Fusarium graminearum*, the PX domain protein regulates the transport of phosphatidylinositol (PI) on vacuolar membranes, mediating toxin secretion (e.g. DON) and invasive hyphae expansion [50]. In summary, FAs and lipid metabolism in vacuoles regulate energy supply, virulence factor synthesis, membrane transport, and signal transduction through multidimensional mechanisms, becoming a central regulatory node in the pathogenic network of filamentous pathogenic fungi. Disruption of this dynamic balance significantly diminishes the host adaptability and pathogenicity of fungi.

FAs also play significant roles in other organelles, such as the endoplasmic reticulum (ER). The ER primarily functions in protein folding and modification, lipid synthesis, calcium storage, and in responding to cellular signals (such as the unfolded protein response, UPR) and various cellular stresses [51]. Additionally, the ER engages in non-vesicular lipid transport with other organelles (e.g. mitochondria, plasma membrane, Golgi apparatus, lipid droplets) via membrane contact sites (MCSs). As the central hub for cellular lipid metabolism, the ER’s core role in mediating lipid exchange at these contact sites through lipid transfer proteins (LTPs) has been highlighted [52]. Elo1 and its interacting proteins Ifa38, Phs1, and Tsc13 form complexes on the ER that regulate the biosynthesis of VLCFAs [53]. Similarly, in *Fusarium graminearum*, FgElo2 is an elongase involved in VLCFA synthesis. Since VLCFA synthesis occurs in the ER, FgElo2 function is directly linked to ER lipid metabolism activities, and its deletion impairs membrane integrity and sensitivity to fungicides [54]. For monounsaturated fatty acid synthesis, *Candida albicans* Spt23p controls the expression of the Δ9 fatty acid desaturase Ole1p. Ole1 localizes to and acts on the ER membrane, catalyzing the synthesis of monounsaturated fatty acids [55]. MoAcb1 (an acyl-CoA-binding protein) participates in the regulation of lipid metabolism. Given that acyl-CoA is a key substrate for lipid synthesis and its metabolism primarily occurs in the ER, MoAcb1 function is closely associated with the ER [16]. Furthermore, MoLfa1 interacts with the ER protein MoMip11, regulating the Mps1-MAPK signaling pathway and affecting hyphal growth, conidial morphology, and infection capability [56]. Compared to studies on the endoplasmic reticulum, investigations into the role of fatty acids in the Golgi apparatus within filamentous pathogenic fungi remain relatively limited.

## FA metabolism in fungal development, stress adaptation, and host interaction

### The effect of FA metabolism for the growth and development of filamentous fungi

FA metabolism is crucial for the growth and development of filamentous fungi, providing essential support for cellular structure and function, and influencing growth processes through the regulation of energy metabolism and signaling pathways. In filamentous fungi, the synthesis, degradation, and modification of FAs are tightly coordinated to meet the demands of different growth stages. For instance, during the germination of conidia and the extension of hyphae, FA metabolism supplies the necessary materials for cell membrane formation and energy metabolism [57]. Lipid droplets inside the cells reserve the majority of the lipids to be used to create the plasma membrane and to be metabolized during conidia formation [58]. In addition to being reserved within lipid droplets and bound to the cell membrane, free FAs such as stearic acid, palmitic acid, oleic acid, and linoleic acid are present within the conidia of *B. bassiana* [12]. The free FAs mobilize endogenous nutrients during the germination of the spores and are precursors to the formation of the cell membrane skeleton [59].

Mutations in the genes essential to the pathway of FA metabolism are often responsible for defects in the growth and development of the fungus. As an example, the absence of the Mfe2 multifunctional enzyme within the smut fungus *U. maydis* in diminished hyphal growth on the long-chain FAs and dramatically reduced virulence [60]. Also, unsaturated FAs and their derivatives are proven to be essential signaling molecules during the formation of the spores within *Aspergillus spp*. The desaturation machinery is exemplified by δ9-stearic desaturase encoded by *sdeA* and *sdeB*, which catalyze the conversion of saturated FAs (16:0 palmitic acid and 18:0 stearic acid) to their monounsaturated counterparts (16:1 palmitoleic acid and 18:1 oleic acid). This enzymatic activity explains the auxotrophic requirement for exogenous oleic acid in minimal fungal growth media [61]. Furthermore, three FA oxygenases (PpoA-C) have been characterized as key regulators coordinating both sexual and asexual reproductive cycles in *A. nidulans* [62–64]. The developmental processes are further modulated by ArfB through N-terminal fatty acylation-dependent control of hyphal polarization [65]. Recent studies in *M. oryzae* revealed the indispensable role of peroxisomal CoA synthetase MoPcs60 in FA metabolism, where its deletion not only disrupts long-chain FA utilization but also compromises pathogenic growth during host infection [66]. Collectively, these findings underscore the centrality of FA metabolic pathways in orchestrating critical developmental and adaptive processes in filamentous fungi.

In terms of applications, the modification and regulation of FA metabolic pathways are of significant importance in industrial applications. Research has demonstrated that modifying FA metabolic pathways can produce high-value compounds such as naringenin and biodiesel [67]. Through genetic engineering, the overexpression of mitochondrial malic enzyme (mME) has successfully increased the yield of polyunsaturated FAs (PUFAs) in oleaginous fungi like *M. alpina* [68]. *A. nidulans* cutinase ANCUT1 and 2 are carboxylate hydrolases with biocatalytic potential. ANCUT2 showed high activity against medium-long chain triacylglycerol and FAs [69]. ANCUT1 has high activity against medium-chain esters and hydroxylated FAs and is capable of esterifying hydroxycinnamic acid to produce compounds with antioxidant properties [70]. These findings provide a basis for the application of ANCUT1 and 2 in biocatalysis processes. In terms of FA synthesis, by disrupting the acyl-CoA synthetase gene in *A. oryzae*, researchers successfully increased the yield of free FAs. This result not only provides new ideas for biodiesel production but also provides an example for genetically engineering fungal metabolic pathways to improve the yield of target products [71]. This metabolic engineering strategy not only enhances the growth performance of fungi but also opens new pathways for the production of high-value biofuels and nutritional supplements.

### Regulation of fungal stress resistance by FA metabolism

FA metabolism has an important role to play in the stress resistance of the filamentous fungus under nutritional limitation and oxidative stress. The pathway of β-oxidation of FAs in the peroxisomes yields acetyl-CoA that supports the growth of the fungus under nutritional limitation through the glyoxylate cycle [17]. Certain FAs such as capric acid and caprylic acid are also shown to inhibit the pathogenicity through the prevention of the pathogen *Candida albicans*’ morphogenesis and biofilm formation [72].

On the antioxidant side, crucial enzymes and signaling molecules within the FA metabolic pathways are essential. As a case in point, in the rice blast fungus *M. oryzae*, the role of the endoplasmic reticulum autophagy (ER-phagy) is played through the acyl-CoA binding protein (MoAcb1), promoting the fungus’s survival under oxidative stress [16]. However, mutations in genes that are included within the FA metabolic pathways generally increase the sensitivity of the fungus to external stress. As an illustration, within the entomopathogenic fungus *B. bassiana*, the deletion of the acyl-CoA synthetase (BbAcs2) causes the germination of the spores to be significantly reduced along with the virulence [73].

FA metabolism is thus not only vital to the adaptability of fungi to environmental stress but also provides a theoretical framework to devise new means to manage fungi. For example, targeting key enzymes in FA metabolic pathways, such as phospholipid methyltransferase (ChoC), is considered a potential strategy for developing novel antifungal agents [21]. Future research will be required to explore further the specific mechanisms of FA metabolism during the stress resistance of fungi and to integrate genetic editing and metabolic engineering techniques to produce more efficient antifungal drugs and biological control strategies.

### Fatty acid-driven lipid droplet dynamics orchestrate nutrient assimilation, membrane homeostasis, and immune evasion in pathogenic fungi

Lipid droplet dynamics are central to *Beauveria bassiana’s* adaptation to insect hosts, directly governing nutrient acquisition and virulence. These droplets facilitate infection through dual mechanisms: serving as lipid conversion hubs that process host lipids into fungal energy and membrane components, and mediating host immune interference by degrading immune signaling molecules to suppress melanization. Specifically, MARVEL domain proteins significantly enhance fungal colonization by maintaining cell membrane integrity and nutrient transport functions. The gene deletion mutants exhibit substantially reduced host cuticle penetration efficiency and fungal load [74]. The lipid droplet assembly protein BbPlin1 acts as a scaffold regulating lipolysis. Its deletion causes abnormal lipid droplet structure and hyphal morphological defects, yet only mildly attenuates virulence, suggesting compensatory metabolic pathways exist [75]. The phospholipase A2 secreted by *B. bassiana* (BbPLA2) hydrolyzes phospholipids in the insect host, releasing free fatty acids, including arachidonic acid (AA) [14]. AA serves as the precursor for the eicosanoid signaling pathway, and its dysregulation significantly suppresses the expression of host immune genes [76]. Specifically, infection with BbPLA2-overexpressing strains caused significant downregulation of insect Toll pathway genes, prostaglandin synthase genes, and 12 antimicrobial peptide (AMP) genes. In contrast, infection with BbPLA2-deficient strains triggered upregulation of these genes. Concurrently, BbPLA2 promotes the accumulation of fungal lipid droplets (LDs), which store hydrolyzed fatty acids to reduce free AA exposure, thereby impairing host immune recognition. Furthermore, during proliferation within the hemocoel, the BbPLA2-overexpressing strain consumed host hemocytes (reducing their number by 20%-38%) and accelerated cuticle penetration, synergistically facilitating immune evasion [15]. Therefore, BbPLA2-mediated fatty acid metabolic derivatives constitute a key mechanism for suppressing host immunity. Comparable observations have been noted in additional pathogenic filamentous fungi. *Verticillium dahlia* phospholipase VdPLP possess a PLA2-like activity, is required for hyphal growth, conidiation, and virulence [77]. Conversely, deletion of sterol hydrolase BbSay1 disrupts lipid droplet distribution and membrane homeostasis, resulting in delayed conidial germination, reduced secretion of cuticle-degrading enzymes, and ultimately severe impairment of virulence [78]. These findings elucidate the pathogenic cascade of *B. bassiana*: host lipid hydrolysis mediated by BbPLA₂, followed by energy storage in droplets facilitated by BbPlin1, maintenance of membrane integrity through MARVEL/BbSay1, and ultimately disruption of host immunity. Notably, virulence contributions vary significantly among these proteins (e.g. minimal impact by BbPlin1), indicating functional redundancy in lipid droplet regulation. This insight informs targeted lipid metabolism strategies for biocontrol development.

### Fatty acid metabolites as mediators of fungal-host immune cross-talk

Fatty acids and their derivatives play a pivotal bidirectional regulatory role in the interactions between pathogenic fungi and host immunity. Pathogenic fungi synthesize oxylipins including eicosanoids, jasmonate-like compounds, and prostaglandins through the lipoxygenase pathway [79,80]. Eicosanoids suppress animal host immunity by inhibiting macrophage phagocytic activity and disrupting leukocyte chemotaxis [80], while oxylipins (e.g. jasmonate analogs) evade host immune responses in plants by interfering with defense hormone signaling networks (e.g. JA/SA balance) and suppressing defense gene expression [81,82]. Simultaneously, fungal-secreted phospholipases hydrolyze host lipid membranes to compromise structural integrity and facilitate tissue invasion [14,15,77]. Fungal-derived oxylipins (e.g. prostaglandins) also regulate their own hyphal differentiation, sporulation, and infection structure development by mimicking host signaling molecules [79].

In host responses, fungal membrane components sphingolipids and glycerolipids are released as signaling molecules during interactions. These molecules act as microbe-associated molecular patterns (MAMPs) detected by plant pattern recognition receptors (PRRs), triggering pattern-triggered immunity (PTI) [83,84]. Fungal infection simultaneously induces plants to produce defense signals including jasmonic acid (JA) and oxylipins, activating disease resistance genes [81]. Plant lipid signaling molecules (e.g. sphingolipids) restrict fungal spread by regulating programmed cell death (PCD) [82]. Collectively, fatty acid metabolites are central to pathogenic fungal invasion and host immune responses, and their synthesis and recognition pathways represent potential targets for antifungal therapies or biocontrol agent optimization.

## The role of regulatory network and mediated epigenetics of FAs metabolism in pathogenic fungi virulence

Findings in *B. bassiana* demonstrate that the transcriptional regulator BbHapX governs the expression of Δ9-FA desaturase (Ole1), a critical enzyme for oleic acid biosynthesis that maintains cell membrane integrity and insect infection efficacy [12]. Parallel regulatory mechanisms involve BbYap1, which orchestrates global FA metabolism to modulate fungal virulence [85]. FA-mediated signaling mechanisms are equally critical. The regulatory network MoLrp1-cAMP-MoMsn2-MoDCI1 mediates FA signal sensing and activates the intracellular cAMP signaling pathway to modulate β-oxidation and lipid droplet mobilization, thereby regulating fungal pathogenicity [20]. MoLfa1 participates in LCFA utilization, coordinates septin ring assembly, and may influence the MPS1-MAPK pathway to collectively affect infection structure development [56].

Beyond its significant impact on fungal virulence through gene-level regulation and signal transduction, FA metabolism and its derivatives also influence fungal physiology and pathogenicity via epigenetic regulation. Acetyl-CoA serves as an essential metabolite in FA metabolism and serves as a critical node in epigenetic regulation. Fluctuations in its concentration directly affect histone acetyltransferase activity, thereby regulating gene expression [86]. In yeast models, acetyl-CoA carboxylase (Acc1) catalyzes the initial step of fatty acid synthesis by consuming acetyl-CoA. Inhibition of Acc1 activity leads to intracellular acetyl-CoA accumulation and significantly elevates global acetylation levels of histones H3 and H4, confirming that Acc1 indirectly regulates epigenetic modifications by modulating the acetyl-CoA pool [87]. The yeast AMPK homolog Snf1 further inhibits Acc1 activity through phosphorylation, increasing acetyl-CoA levels and promoting histone H3 acetylation. Conversely, Snf1 deletion reduces acetyl-CoA availability and histone acetylation, indicating that the Snf1-Acc1 axis maintains epigenetic regulation by stabilizing acetyl-CoA homeostasis [88]. AMPK/Snf1 signaling also influences histone acetylation by coordinately regulating acetyl-CoA synthetase and carboxylase activities. AMPK activation increases acetyl-CoA production and suppresses fatty acid synthesis, ultimately altering specific gene expression [89]. This metabolism-epigenetics coupling mechanism participates in cellular stress adaptation. Mutations in fission yeast fatty acid synthase cause acetyl-CoA accumulation and localized chromatin hyperacetylation, activating oxidative stress-responsive genes and enhancing cellular resistance to H₂O₂ [90].

In the pathogenic fungus *Candida albicans*, the exogenous short-chain fatty acid crotonate increases H3 crotonylation. This downregulates hypha-specific genes (e.g. HWP1, ECE1), thereby suppressing hyphal formation and reducing host cell damage and immune evasion [91]. However, studies on other pathogenic fungi remain scarce. Further investigation is warranted to elucidate how FA metabolism-mediated epigenetic regulation globally modulates gene expression and virulence of pathogenic fungi.

## Summary and outlook

**Figure 1.**
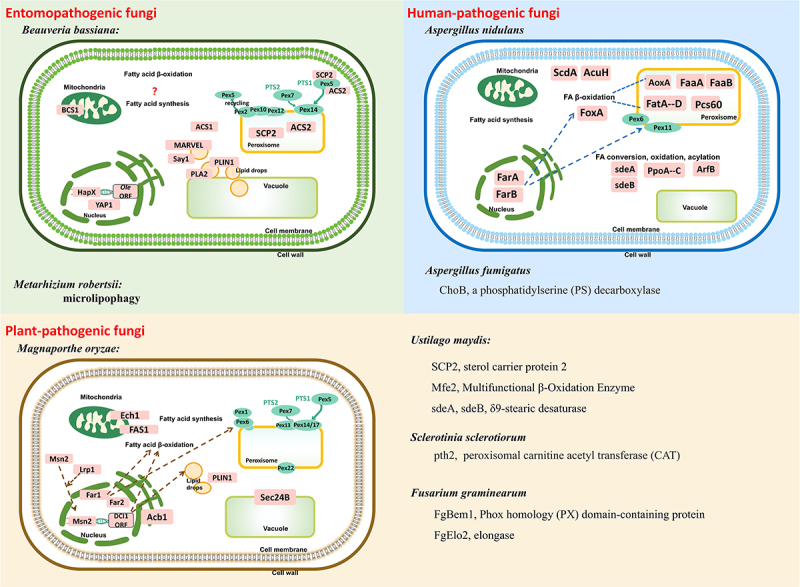
Functional gene landscape of FA metabolism in filamentous pathogenic fungi.

FAs metabolism serves as a central regulatory hub governing the pathogenicity and host adaptation of filamentous pathogenic fungi. As shown in Figure 1, species-specific adaptations of key regulators highlight conserved yet diversified regulatory branches within FA networks. Collective information listed in Table 1 jointly offers reference for interventions targeting novel antifungal strategies and the enhancement of entomopathogenic fungal virulence.

**Table 1.** Summary of the biological functions of FAs metabolism functional genes in filamentous pathogenic fungi.

Fungi	Gene	Biological function	Linked fungal phenotype			Reference
***Human-pathogenic fungi***						
*Aspergillus nidulans*	*ScdA* (acyl-CoA dehydrogenase)	short-chain FAs β-oxidation	growth		20	
	*acuH* (carnitine/acylcarnitine carrier)	long-chain FA and acetic acid metabolism	growth		25	
	*FarA/FarB (Far1/Far2)* (transcription factors)	synchronize β-oxidation (*MFP1, FOXA*) and peroxisome biogenesis (*PEX6, PEX11*)	conidial germination and host colonization		37, 38	
	*AoxA* (long-chain fatty acyl-CoA oxidase)	FA degradation	growth		39	
	*FatA-D, FaaA-B* (fatty acyl-CoA synthetases)	FA degradation	growth		40	
	*ArfB* (ADP ribosylation factors)	vesicle assembly and trafficking	polarized growth		65	
	*PpoA-C* (three FA oxygenases)	biosynthesis of the linoleic-acid-derived psi (precocious sexual inducer) factor component		sexual and asexual development	62–64	
	*ANCUT1/2* (Cutinase)	split carboxylic acid ester		*unreported*	69, 70	
*Aspergillus fumigatus*	*ChoC* (phospholipid methyltransferase)	addition of methyl groups to phosphatidylethanolamine (PE)		cell membrane integrity and reduces pathogenicity	19	
***Entomopathogenic fungus***						
*Beauveria bassiana*	*BbBCS1* (mitochondrial AAA protein)	maintenance of mitochondrial function and FA homeostasis		growth and virulence	24	
	*BbSCP2* (sterol carrier protein 2)	nonspecific lipid transport		conidial germination and virulence	43	
	*BbHapX* (basic leucine zipper (bZIP) transcription factor)	orchestrates global FA metabolism, fungal adaptation to iron availability		conidial UFA storage and virulence	12	
	*BbOle1* (Δ9-FA desaturase)	oleic acid biosynthesis		virulence	12	
	*BbYap1* (bZIP transcription factor, yeast activator protein)	orchestrates global FA metabolism		growth and virulence	87	
	*BbMARVEL1–5*	maintaining the lipid-droplet homeostasis		growth and virulence	74	
	*BbPlin1* (perilipin)	lipid droplet structural maintenance and turnover		growth and virulence	75	
	*BbPLA2* (phospholipase A2)	lipid acquisition from the host		virulence	78	
	*BbSay1* (sterol acetylhydrolase)	lipid droplet accumulation		growth and virulence	80	
	*BbAcs2* (acyl-CoA synthetase)	catalyzing acetate and CoA into acetyl-CoA		germination and virulence	73	
***Plant-pathogenic fungi***						
*Magnaporthe oryzae*	*MoAcb1* (acyl-CoA binding protein)	bind both medium-chain and long-chain acyl-CoA esters		conidiation, appressorium development, and pathogenicity	14	
	*Ech1* (enoyl-CoA hydratase)	involved in mitochondrial β-oxidation pathway		germination and virulence	17	
	*MoMsn2* (transcription factor)	regulating expression of *MoDCI1*		invasive hyphal growth	18	
	*MoDCI1* (dienoyl-CoA isomerase)	modulating β-oxidation flux and lipid droplet mobilization		infectious growth		
	*MoLrp1* (low-density lipoprotein receptor-related protein)	sensing fatty acid signal and activates the intracellular cAMP signaling pathway to induce nuclear accumulation of MoMsn2		infectious growth		
	*Elo1* (fatty acid elongase)	biosynthesis of VLCFAs		pathogenicity	52	
	*MoLfa1* (hypothetical protein involed in LCFAs utilization)	interacts with endoplasmic reticulum protein MoMip11 to regulate the Mps1-MAPK signaling pathway		mycelia growth, conidial morphology, and infection ability	55	
	*MoPcs60* (CoA synthetase)	FA metabolism		pathogenic growth during host infection	65	
*Ustilago maydis*	*Mfe2* (multifunctional enzyme type 2)	multifunctional enzyme in β-oxidation of FAs		growth, virulence	59	
	*SCP2* (sterol carrier protein 2)	nonspecific lipid transport		conidial germination and virulence	44	
	*sdeA*, *sdeB* (δ9-stearic desaturase)	conversion of saturated FAs to their monounsaturated counterparts		fungal growth	60	
*Sclerotinia sclerotiorum*	*Pth2* (carnitine acetyltransferase)	oxalic acid synthesis.		host colonization	15	
*Fusarium graminearum*	*FgBem1* (PX domain-containing protein)	transport of PI on vacuolar membranes		toxin secretion, invasive hyphae expansion	49	
	*FgElo2* (elongase)	involved in VLCFA synthesis		virulence	53	
*Verticillium dahliae*	*VdPLP* (patatin-like phospholipase)	catalyze the cleavage of fatty acids from membrane lipids		hyphal growth, conidiation, and virulence	78	

Despite these advances, critical knowledge gaps persist in our understanding of FA metabolism within filamentous pathogenic fungi. Firstly, the interplay between fungal FA metabolites and host immune responses, particularly how FA derivatives influence immune recognition or evasion, needs further characterized. Secondly, molecular mechanisms underlying FA synthesis and trafficking in the endoplasmic reticulum and Golgi apparatus require systematic exploration. Thirdly, the mechanisms associated with FA metabolism-mediated epigenetic regulation in filamentous pathogenic fungi virulence warrant strengthened investigation. To address these gaps, future research should leverage integrated multi-omics platforms, super-resolution imaging technologies, and advanced gene editing systems. These approaches are essential to unravel the spatiotemporal orchestration of FA metabolism during critical host-pathogen interactions, particularly elucidating the stage-specific FA metabolic reprogramming that drives infection progression.

## Data Availability

Data availability is not applicable to this article as no new data were created or analyzed in this study.
