# Correction

**DOI:** 10.1080/21505594.2025.2566547

**Published:** 2025-10-06

**Article title**: Fatty acids in filamentous pathogenic fungi: Multidimensional regulation and pathogenic mechanisms

**Authors**: Haiyan Lin and Yuejin Peng

**Journal**: *VIRULENCE*

**DOI**: http://dx.doi.org/ 10.1080/21505594.2025.2561826

In the originally published article, there is a discrepancy in the citation numbers in Table 1. This has now been corrected, and the article has been republished online.

**Table 1.** Summary of the biological functions of FAs metabolism functional genes in filamentous pathogenic fungi.

Fungi	Gene	Biological function	Linked fungal phenotype		Reference
***Human-pathogenic fungi***					
*Aspergillus nidulans*	*ScdA* (acyl-CoA dehydrogenase)	short-chain FAs β-oxidation	growth	22	
	*acuH* (carnitine/acylcarnitine carrier)	long-chain FA and acetic acid metabolism	growth	27	
	*FarA/FarB (Far1/Far2)* (transcription factors)	synchronize β-oxidation (*MFP1, FOXA*) and peroxisome biogenesis (*PEX6, PEX11*)	conidial germination and host colonization	38, 39	
	*AoxA* (long-chain fatty acyl-CoA oxidase)	FA degradation	growth	40	
	*FatA-D, FaaA-B* (fatty acyl-CoA synthetases)	FA degradation	growth	41	
	*ArfB* (ADP ribosylation factors)	vesicle assembly and trafficking	polarized growth	65	
	*PpoA-C* (three FA oxygenases)	biosynthesis of the linoleic-acid-derived psi (precocious sexual inducer) factor component	sexual and asexual development	62-64	
	*ANCUT1/2* (Cutinase)	split carboxylic acid ester	*unreported*	69, 70	
*Aspergillus fumigatus*	*ChoC* (phospholipid methyltransferase)	addition of methyl groups to phosphatidylethanolamine (PE)	cell membrane integrity and reduces pathogenicity	21	
***Entomopathogenic fungus***					
*Beauveria bassiana*	*BbBCS1* (mitochondrial AAA protein)	maintenance of mitochondrial function and FA homeostasis	growth and virulence	26	
	*BbSCP2* (sterol carrier protein 2)	nonspecific lipid transport	conidial germination and virulence	44	
	*BbHapX* (basic leucine zipper (bZIP) transcription factor)	orchestrates global FA metabolism, fungal adaptation to iron availability	conidial UFA storage and virulence	12	
	*BbOle1* (Δ9-FA desaturase)	oleic acid biosynthesis	virulence	12	
	*BbYap1* (bZIP transcription factor, yeast activator protein)	orchestrates global FA metabolism	growth and virulence	85	
	*BbMARVEL1-5*	maintaining the lipid-droplet homeostasis	growth and virulence	74	
	*BbPlin1* (perilipin)	lipid droplet structural maintenance and turnover	growth and virulence	75	
	*BbPLA2* (phospholipase A2)	lipid acquisition from the host	virulence	15	
	*BbSay1* (sterol acetylhydrolase)	lipid droplet accumulation	growth and virulence	78	
	*BbAcs2* (acyl-CoA synthetase)	catalyzing acetate and CoA into acetyl-CoA	germination and virulence	73	
***Plant-pathogenic fungi***					
*Magnaporthe oryzae*	*MoAcb1* (acyl-CoA binding protein)	bind both medium-chain and long-chain acyl-CoA esters	conidiation, appressorium development, and pathogenicity	16	
	*Ech1* (enoyl-CoA hydratase)	involved in mitochondrial β-oxidation pathway	germination and virulence	19	
	*MoMsn2* (transcription factor)	regulating expression of *MoDCI1*	invasive hyphal growth	20	
	*MoDCI1* (dienoyl-CoA isomerase)	modulating β-oxidation flux and lipid droplet mobilization	infectious growth		
	*MoLrp1* (low-density lipoprotein receptor-related protein)	sensing fatty acid signal and activates the intracellular cAMP signaling pathway to induce nuclear accumulation of MoMsn2	infectious growth		
	*Elo1* (fatty acid elongase)	biosynthesis of VLCFAs	pathogenicity	53	
	*MoLfa1* (hypothetical protein involed in LCFAs utilization)	interacts with endoplasmic reticulum protein MoMip11 to regulate the Mps1-MAPK signaling pathway	mycelia growth, conidial morphology, and infection ability	56	
	*MoPcs60* (CoA synthetase)	FA metabolism	pathogenic growth during host infection	66	
*Ustilago maydis*	*Mfe2* (multifunctional enzyme type 2)	multifunctional enzyme in β-oxidation of FAs	growth, virulence	60	
	*SCP2* (sterol carrier protein 2)	nonspecific lipid transport	conidial germination and virulence	45	
	*sdeA, sdeB* (δ9-stearic desaturase)	conversion of saturated FAs to their monounsaturated counterparts	fungal growth	61	
*Sclerotinia sclerotiorum*	*Pth2* (carnitine acetyltransferase)	oxalic acid synthesis.	host colonization	17	
*Fusarium graminearum*	*FgBem1* (PX domain-containing protein)	transport of PI on vacuolar membranes	toxin secretion, invasive hyphae expansion	50	
	*FgElo2* (elongase)	involved in VLCFA synthesis	virulence	54	
*Verticillium dahliae*	*VdPLP* (patatin-like phospholipase)	catalyze the cleavage of fatty acids from membrane lipids	hyphal growth, conidiation, and virulence	77	

